# Dynamic flux balance modeling to increase the production of high-value compounds in green microalgae

**DOI:** 10.1186/s13068-016-0556-4

**Published:** 2016-08-04

**Authors:** Robert J. Flassig, Melanie Fachet, Kai Höffner, Paul I. Barton, Kai Sundmacher

**Affiliations:** 1 Max Planck Institute for Dynamics of Complex Technical Systems, Process Systems Engineering, Sandtorstr.1, 39106 Magdeburg, Germany; 2 Massachusetts Institute of Technology, Process Systems Engineering, Cambridge, MA 02139 USA; 3 Otto-von-Guericke-University Magdeburg, Process Systems Engineering, Universitätsplatz 2, 39106 Magdeburg, Germany

**Keywords:** *Dunaliella salina*, $\upbeta$-carotene, Dynamic flux balance analysis, Model-based process design

## Abstract

**Background:**

Photosynthetic organisms can be used for renewable and sustainable production of fuels and high-value compounds from natural resources. Costs for design and operation of large-scale algae cultivation systems can be reduced if data from laboratory scale cultivations are combined with detailed mathematical models to evaluate and optimize the process.

**Results:**

In this work we present a flexible modeling formulation for accumulation of high-value storage molecules in microalgae that provides quantitative predictions under various light and nutrient conditions. The modeling approach is based on dynamic flux balance analysis (DFBA) and includes regulatory models to predict the accumulation of pigment molecules. The accuracy of the model predictions is validated through independent experimental data followed by a subsequent model-based fed-batch optimization. In our experimentally validated fed-batch optimization study we increase biomass and $\upbeta$-carotene density by factors of about 2.5 and 2.1, respectively.

**Conclusions:**

The analysis shows that a model-based approach can be used to develop and significantly improve biotechnological processes for biofuels and pigments.

## Background

Exploration of marine and aquatic microorganisms for the development of sustainable bioprocesses has received renewed interest in recent years due to a diverse range of biochemical products that can be synthesized, making algae an attractive target for bioprocess design. Usually in photobiotechnology low biomass densities and product contents are achieved. Therefore biorefinery approaches have been proposed to exploit the economic potential of the process fully by not only considering the main product but also important by-products.

Algae of the genus *Dunaliella* are important production organisms for high-value pigments, biofuels and platform chemicals. *D. salina*  is a halotolerant green microalga especially found in saline shallow lakes. The high salt tolerance of *D. salina*  is one of the key distinguishing features of this organism. It accumulates $\upbeta$-carotene in response to abiotic stress. Of all influencing factors, nitrogen depletion and high photon flux density strongly favor $\upbeta$-carotene accumulation in *D. salina*  [1]. An understanding of microalgal metabolism is necessary to optimize the production yield. In particular, there is a need for a model formulation that provides predictions of valuable microalgal biomass fractions such as pigments and triacylglycerides. One promising modeling framework is based on dynamic flux balance analysis (DFBA). A DFBA model formulation is the integration of genome-scale metabolic models with mass conservation laws applied to the extracellular environment [2]. The current work presents an extension of the DFBA formulation to stress-induced $\upbeta$-carotene accumulation in *D. salina*. The objective of this work is to develop and validate a detailed and structured model for *D. salina*  with focus on quantitative prediction of stress-induced $\upbeta$-carotene production dependent on different external stimuli. Existing modeling formulations for dynamic prediction of microalgal metabolism focus on lipid production, as a feedstock for the next generation of biofuels, and do not include stress-induced accumulation of other metabolites. DRUM (Dynamic Reduction of Unbalanced Metabolism) is a dynamic metabolic modeling framework which includes accumulation of intracellular metabolites [3]. This approach relies on the definition of subnetworks and elementary flux mode (EMF) analysis of each of the subnetworks. Since EFM analysis can become computationally expensive, this may not be applicable to large (genome-scale) networks. Furthermore, prior knowledge regarding the division into subnetworks must be available and kinetic expressions have to be assigned to each of the subnetworks. In contrast, the current formulation does not require EFM and therefore can be applied to genome-scale networks. Another approach that has been presented recently is MetDFBA [4]. This extension of DFBA assumes that metabolomics measurements are available. Such an assumption may not be feasible for large-scale production systems. The derivation of the model presented in this paper also takes advantage of existing detailed metabolic models. The development of genome-scale metabolic network reconstructions for microalgae have been recently reviewed in [5]. The remainder of this paper is structured as follows. The experimental setup and analysis methods are described in “Methods” section. In “Results” section, the extended DFBA model is first developed, then the results of parameter estimation and the validation of the model are discussed. The paper concludes with a summary of the results and discussion of future research.

## Methods

### Cultivations in flat-plate photobioreactors in batch operation mode

Strain, growth medium, pre-cultivation and analytical methods are as described in [6]. The experimental data for the model calibration was collected from cells grown in a 1 L flat-plate photobioreactor (FMT150, Photon Systems Instruments, 5 cm path length), referred to as the PSI reactor, illuminated with white and red LEDs. Subsequently, the model was validated using cells cultivated in 1.8 L flat-plate photobioreactor (Labfors Lux, Infors HT, 2 cm path length), referred to as the INFORS reactor, equipped with warm white LEDs. Both reactors were aerated with $\mathrm {CO}_2$-enriched air (97 % air and 3 % $\mathrm {CO}_2$) using a flow rate of 500 mL min$^{-1}$ adjusted by mass flow controllers. The pH was maintained at 7.5 by automated addition of 1M HCl and 1M KOH. Temperature was maintained at 24 $^\circ$C. The inoculation of the reactor was performed from a stock culture grown under low light and nitrogen-replete conditions.

### Pigment extraction and detection using high performance liquid chromatography (HPLC)

The pigment extraction was performed according to [7]. The $\upbeta$-carotene, chlorophyll *a* and *b* fractions of the cells were determined using HPLC (Agilent 1100, Agilent Technology, USA) equipped with a Reversed-Phase C18 column (Zorbax Eclipse Plus, 1.8 $\upmu$m pore size) and 2 $\upmu$L injection volume. The pigment extract was eluted with a linear gradient from 100 % eluent A [84 % acetonitrile, 2 % methanol, 14 % Tris buffer (0.1 M, pH 8.0)] to 10 % A and 90 % eluent B (68 % methanol, 32 % ethyl acetate) for 2 min followed by elution with 100 % B for 3 min at a flow rate of 0.5 mL min$^{-1}$ and detected with a diode array detector (DAD) [8]. The pigments were identified and quantified by comparing retention time and spectral absorption characteristics with commercial pigment standards (Sigma Aldrich, USA).

### Simulation methods

The numerical solution of the proposed model involves the use of DFBAlab [9], a MATLAB code that performs reliable and efficient DFBA simulations. Widespread implementation of DFBA has been hindered by numerical complications resulting from linear programs (LPs) becoming infeasible and having non-unique solution vectors. Infeasible LPs cause simulation failure as the right-hand side of the ODEs becomes undefined, and non-unique solution vectors cause this same right-hand side to be non-unique, producing an ODE system that integrators are unable to solve. These complications are addressed in [10]. DFBAlab is a modified MATLAB implementation of the simulator in [10] that uses the Phase I LP to avoid infeasibilities and lexicographic optimization to provide unique exchange fluxes. It reformulates the LP locally as an algebraic system, and integrates a differential-algebraic equation system instead of ODEs with LPs embedded to increase speed.

### Parameter estimation

The model parameters were estimated by minimizing the residual sum of squares

1 $$\begin{aligned} \chi ^2(\mathbf {p}) = \sum _{k=1}^m\sum _{i=1}^{d_k} \frac{1}{\sigma _{ki}^2}(y_k(t_i)-\hat{y}_k(t_i,\mathbf {p}))^2, \end{aligned}$$

where *m* is the number of measured outputs, $d_k$ is the number of measurement time points, $y_k$ and $\hat{y}_k$ are the *k*th measured output variable and corresponding model prediction, respectively, and $\mathbf {p}$ is a vector of model parameters. The process output measurements are the biomass concentration, the extracellular nitrogen concentration, the chlorophyll and $\upbeta$-carotene fractions of the biomass. The parameter values were partially taken from literature and derived from experimental data as discussed in the results section.

## Results and discussion

### Dynamic flux balance model

In FBA, models are based on the assumption that the intracellular reaction network has reached a quasi-steady state (balanced-growth assumption). In DFBA, it is assumed that the intracellular dynamics are fast compared to extracellular dynamics such that the quasi-steady state approximation for the FBA model remains valid. For photosynthetic organisms that undergo constant environmental fluctuation this assumption is not justifiable. Indeed, dynamic intracellular accumulation and consumption are essential in the metabolism of the cells. Therefore, in the current formulation, this is modeled by introducing intracellular dynamic states. The DFBA model developed here consists of two main components, a metabolic model of the microalga and a dynamic model of the photobioreactor environment. The dynamic state variables of the model are the extracellular dynamic states, which are the biomass concentration on dry weight basis *x*, and the extracellular nitrate concentration $c_{\mathrm {NO_3}}$, in addition to the intracellular dynamic states, which are the chlorophyll fraction of total biomass $\omega _\mathrm{Chl}$, the $\upbeta$-carotene fraction of total biomass $\omega _\mathrm{Car}$, and the nitrogen cell quota $\omega _\mathrm{N}$.

#### Flux balance model

Although the genome of *D. salina*  has been largely sequenced, a genome-scale metabolic network reconstruction is currently not available. Therefore the genome-scale metabolic network reconstruction of the green fresh water alga *Chlamydomonas reinhardtii* [11, 12] is used to demonstrate the applicability of this network for prediction of the pigment production of *D. salina*  under different abiotic stress conditions. A key aspect of this study is the prediction of light- and nitrogen-dependent growth and production of $$\upbeta$$-carotene. Hence it is important that the $$\upbeta$$-carotene synthesis and biomass yield are comparable between the two species. Comparison between the $$\upbeta$$-carotene synthesis pathways of *C. reinhardtii* and *D. salina*  based on [13] reveals that the pathway is conserved across the two species. The biomass composition for autotrophic growth in the FBA model consists of 176 unique metabolites [11]. The FBA model predicts a steady-state chlorophyll fraction of $$0.76\;{\rm mg}_\mathrm{Car}/{\rm g}_\mathrm{Dw}$$$$\upbeta$$-carotene, and $$24\;{\rm mg}_\mathrm{Chl}/{\rm g}_\mathrm{Dw}$$ ($$9\;{\rm mg}_\mathrm{Chl}/{\rm g}_\mathrm{Dw}$$ chlorophyll *a* and $$15\;{\rm mg}_\mathrm{Chl}/{\rm g}_\mathrm{Dw}$$ chlorophyll *b*) for autotrophic growth under low light and nitrogen-replete conditions. In comparison, the measured $$\upbeta$$-carotene fraction of the inoculum in *D. salina*  was 1.5–$$3\;{\rm mg}_\mathrm{Car}/{\rm g}_\mathrm{Dw}$$, and the experimentally observed total chlorophyll fraction for *D. salina*  was in the range of 10–$$40\;{\rm mg}_\mathrm{Chl}/{\rm g}_\mathrm{Dw}$$. Since the difference between the two values have only little impact on DFBA predictions, the $$\upbeta$$-carotene and chlorophyll fractions were left unchanged. The stoichiometric network of the FBA model is extended to account for the accumulation of chlorophyll and $$\upbeta$$-carotene. In particular, reversible *accumulation fluxes* are added to the mass balance of these metabolites. Their upper and lower bounds are determined by the regulatory models described below. The newly defined fluxes are conceptually the same as the exchange fluxes, except that they describe reversible exchange with an intracellular storage. Here, reversible means that the model can accumulate and consume any of the stored molecules, depending on the result of the FBA.

#### Exchange fluxes

In addition to the modification of the FBA model, the upper and lower bounds on the exchange and accumulation fluxes are specified as follows.

*Light attenuation * A common approach to model the light attenuation in a fluid is based on the Lambert-Beers law. Based on this, the average light intensity in the reactor volume can be estimated as

$$\begin{aligned} \bar{E}&= \frac{1}{L_\mathrm{r}} \int _0^{L_\mathrm{r}} E_0 \exp (-K_ez)\,{\rm d}z,\\&= \frac{E_0}{L_rK_e}\left( 1 - \exp (-K_\mathrm{e}L_\mathrm{r}) \right) , \end{aligned}$$

where $E_0$ is the incident light intensity at the reactor surface, $L_\mathrm{r}$ is the reactor length, and $K_\mathrm{e}$ is the optical depth as a function of the chlorophyll concentration in the bioreactor. The optical depth $K_e$ is determined as the product of the chlorophyll concentration and the average chlorophyll specific absorption coefficient $K_{\mathrm{e},0}$, i.e., $K_\mathrm{e}=K_{\mathrm{e},0}\,\omega _\mathrm{Chl}\,x$. The average experimentally observed optical specific absorption coefficient was determined to be $K_{\mathrm{e},0}=11.5\,{\rm m}^2/{\rm g}_\mathrm{Chl}$, based on [6]. The average biomass specific light intensity that is the input to the FBA model is determined as follows. First, it is assumed that the light energy available for photosynthesis depends on the chlorophyll fraction. Therefore a cell specific efficiency for utilizing the light energy is proposed as an affine function of the chlorophyll concentration with the parameters $\eta _0$ and $\eta _1$, i.e.,

2 $$\begin{aligned} \eta = \theta _\mathrm{eff}(\eta _1 \omega _\mathrm{chl} + \eta _0), \end{aligned}$$

where $\omega _\mathrm{Chl}$ is the chlorophyll fraction of the biomass, $\theta _\mathrm{eff}$ is dimensionless efficiency factor as in [11]. Then the average biomass specific light intensity is given by

$$\begin{aligned} \bar{E}_x= \frac{\theta _\mathrm{dim} \eta }{\rho _\mathrm{A}}\bar{E}, \end{aligned}$$

where $\theta _\mathrm{dim}$ is a unit conversion factor such that the unit of $\bar{E}_\mathrm{x}$ is consistent with existing FBA model input unit, and $\rho _\mathrm{A} = xL_\mathrm{r}$ is the biomass density per surface area of the bioreactor in ${\rm g}_\mathrm{Dw}/{\rm m}^2$. Finally, the upper and lower bound on the light exchange flux are set to the average biomass specific light intensity.

*Nitrate metabolization flux * The rate at which the internal nitrate storage (see Droop model below) is metabolized is denoted by $v_{\mathrm {NO_3,met}}$. As with all other exchange fluxes, the sign convention implies that a negative values indicates a consumption of nitrate. The lower bound on nitrate metabolization rate is given by

$$\begin{aligned} v^{LB}_{\mathrm {NO_3,met}} = -v_{\mathrm {NO_3,met,max}}\left( 1-\frac{\omega _{\mathrm {N},{\rm min}}}{\omega _{\mathrm {N}}}\right) , \end{aligned}$$

where the maximal flux value $v_{\mathrm{NO}_3,\mathrm{met,max}}$ is considered as a model parameter. The metabolization is inhibited as the nitrogen cell quota $\omega _{\mathrm {N}}$ reaches its minimal value $\omega _{\mathrm {N,min}}$. Furthermore, it is assumed that no nitrate is synthesized through this flux and therefore the upper bound of the nitrate metabolization flux is set to zero.

*Chlorophyll accumulation flux * An empirical model for the chlorophyll accumulation is developed based on the observation that the ratio between chlorophyll and nitrogen in various photosynthetic microorganisms can be approximated by a simple inhibition function of the incident light [14, 15]. Specifically, it is assumed that the ratio is given by

$$\begin{aligned} \gamma (\bar{E}) = \gamma _{\mathrm{max}} \frac{K_\mathrm{E}}{\bar{E} + K_\mathrm{E}}, \end{aligned}$$

where $\gamma _{\mathrm{max}}$ is the maximal ratio and $K_\mathrm{E}$ is an inhibition constant. Furthermore, since the data in [14] is collected under low and medium light conditions, it is assumed that the ratio is constant for irradiance higher than a critical value $\bar{E}_{\mathrm{sat}}$. The synthesis of chlorophyll in the metabolic network is enforced by varying the bounds on the chlorophyll accumulation flux. If the lower bound is greater than zero, only flux distributions with positive chlorophyll accumulation flux are feasible in the FBA model. This redirects part of the metabolic activity towards chlorophyll synthesis. Hence, to model the regulation of chlorophyll synthesis the lower bound on the chlorophyll flux is assumed to be of the form

$$\begin{aligned} v^{\mathrm{LB}}_{\mathrm{Chl}} = \left( \gamma (\bar{E}) - \frac{\omega _{\mathrm{Chl}}}{\omega _\mathrm {N}}\right) . \end{aligned}$$

If $\frac{\omega _{\mathrm{Chl}}}{\omega _\mathrm {N}}<\gamma (\bar{E})$, then the lower bound is greater than zero, which enables accumulation of chlorophyll. If $\frac{\omega _{\mathrm{Chl}}}{\omega _\mathrm {\mathrm{N}}}>\gamma (\bar{E})$, then the lower bound is less than zero, which enables metabolization of chlorophyll. This regulation can be considered as a proportional control structure with the aim to track the ratio between chlorophyll and nitrogen. The analogy between biochemical regulation and control structures is illustrated in detail in [16]. The maximal chlorophyll to nitrogen ratio $\gamma _{\mathrm{max}}$, the inhibition constant $K_\mathrm{E}$, and the saturation light intensity are model parameters. $\upbeta$*-carotene exchange flux:*$\upbeta$-carotene is produced in algae to increase the dissipation of energy at high photon flux densities [17]. Under these conditions the microorganisms cannot utilize all light energy in the photosystem and the excess is harmful to the cell. Furthermore, the mechanism is “energetically preferable” only if the amount of available nitrogen is low. If sufficient nitrogen (and carbon) is available, then the light energy is used to generate more biomass. Therefore, the model for synthesis of $\upbeta$-carotene is based on the following line of reasoning. First, it is assumed that for high photosynthesis rates, which are measured by average biomass specific light intensity, the biosynthesis of $\upbeta$-carotene as a secondary carotenoid and energy sink is induced, while for low photosynthesis rates no accumulation occurs. The lower bound on this light induced $\upbeta$-carotene flux is modeled via a Hill function

$$\begin{aligned} v^{\mathrm{LB}}_{\mathrm{Car, gen}} = v_{\mathrm{Car,max}} \frac{\bar{E}_\mathrm{x}^{n_{\mathrm{Car}}}}{ \bar{E}_{\mathrm{x,A}}^{n_{\mathrm{Car}}} + \bar{E}_x^{n_{\mathrm{Car}}} }. \end{aligned}$$

The Hill exponent $n_{\mathrm{Car}}$, the critical average light intensity on biomass $\bar{E}_{\mathrm{x,A}}$ required for $\upbeta$-carotene synthesis, and the maximal $\upbeta$-carotene flux $v_{\mathrm{Car,max}}$ are considered as model parameters. In addition to the light dependent regulation, the dependency on intracellular nitrogen availability has to be taken into account. At high nitrogen quota, the $\upbeta$-carotene synthesis rate is down regulated since sufficient nitrogen is available for biomass synthesis. Hence, the light induced $\upbeta$-carotene flux is only active if the following simple relationship between nitrogen cell quota and the average biomass specific light intensity is satisfied:

$$\begin{aligned} \omega _\mathrm{N} \le \alpha _1 \bar{E}_\mathrm{x} + \alpha _0. \end{aligned}$$

**Fig. 1:**
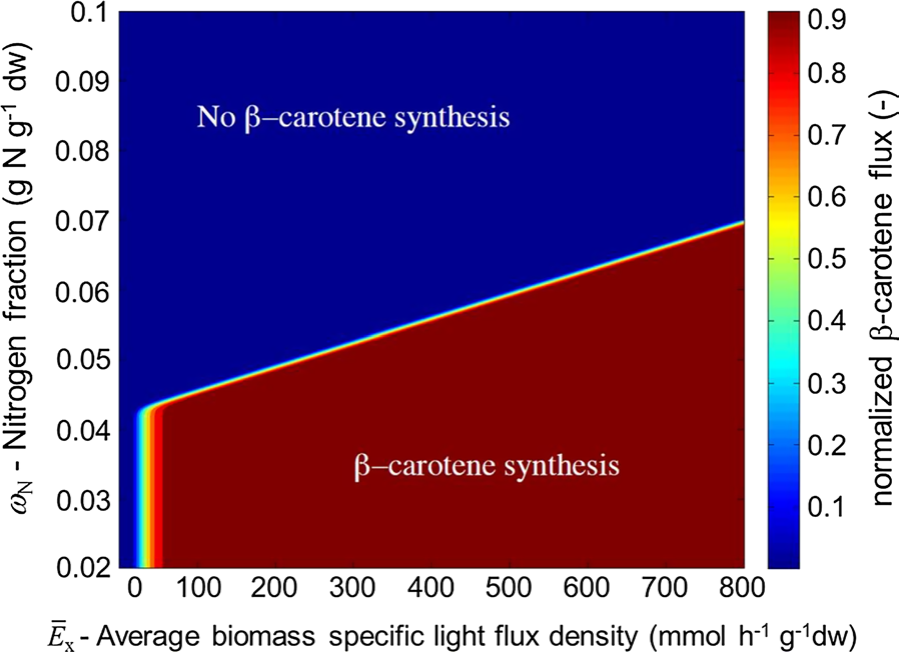
Heat map of the $\upbeta$-carotene synthesis rate: The assumed relationship between nitrogen quota, light intensity and $\upbeta$-carotene synthesis is illustrated

This implies that at low nitrogen cell quota, $\upbeta$-carotene synthesis is induced at low light intensities. Combining these two mechanisms yields that the lower bound of the $\upbeta$-carotene exchange flux in the FBA model is given by

3 $$v_{\mathrm{Car}}^{{\mathrm{LB}}} = v_{\mathrm{Car, gen}} \Psi (\alpha _1\bar{E}_\mathrm{x} + \alpha _0 - \omega _\mathrm{{N}}),$$

where $\Psi (\mathrm{} x,\eta _\mathrm{s})=1/(1+e^{(-\eta _\mathrm{}s x)})$ is a smoothing function with smoothing factor $\eta _\mathrm{s}$ such that lower bound is a smooth function of the dynamic states. Similar to the chlorophyll flux, a positive value for this lower bound implies the accumulation of $\upbeta$-carotene. The upper bound on the $\upbeta$-carotene accumulation flux is set to be infinite. A graphical illustration of the dependency of the $\upbeta$-carotene synthesis on the nitrogen cell quota and average biomass specific light intensity is shown in Fig. 1.

*Lag phase * After inoculation, the cells first adapt to the conditions in the photobioreactor, this lag phase is modeled as a Hill function $\psi$ given by

$$\begin{aligned} \psi = \frac{t^{n_{\mathrm{lag}}}}{\lambda ^{n_{\mathrm{lag}}} + t^{n_{\mathrm{lag}}}}, \end{aligned}$$

where $\lambda$ is the duration of the lag phase in days, $n_{\mathrm{lag}}$ is the Hill coefficient, and *t* is time in days. The duration of the lag phase is considered as a model parameter and the Hill coefficient is fixed to $n_{\mathrm{lag}}=4$ according to [6]. The upper and lower bounds of all exchange fluxes including the flux for non-growth associated ATP maintenance are multiplied by the Hill function $\psi$ such that all metabolic activities are effected during the lag phase.

#### Dynamic photobioreactor model

The flat-plate photobioreactor environment is assumed to be well-mixed such that there are no gradients in nutrients or biomass concentration in the reactor volume. The mass balance for nitrate as an extracellular metabolite is explicitly included in the reactor model. Dissolved $\mathrm {CO}_2$ is provided in excess and is assumed to be not growth-limiting, hence the exchange flux for $\mathrm {CO}_2$ in the FBA model is unbounded and the $\mathrm {CO}_2$ mass balance is not included in the dynamic model. Furthermore, it is assumed that the dissolved $\mathrm {O}_2$ concentration is constant and at equilibrium with the environment, hence the exchange flux for $\mathrm {O}_2$ is unbounded and the $\mathrm {O}_2$ mass balance is not included in the dynamic model.

*Nitrogen quota*: The Droop model is a widely used formulation to represent limitation of substrate uptake due to internal accumulation [18]. The model promotes the idea that growth depends on the stored intracellular pool of nutrients and not directly on the extracellular concentration. The nitrogen quota $\omega _N$ is described by the following ordinary differential equation

$$\begin{aligned} \dot{\omega }_{\mathrm {N}} = \frac{m_{\mathrm {N}}}{m_{\mathrm {NO_3}}}v_{\mathrm {NO_3}} -\upmu \omega _{\mathrm {N}}, \end{aligned}$$

where $v_{\mathrm {NO_3}}$ in the first term is the nitrate uptake rate determined by Michaelis–Menten kinetics, and $\upmu$ is the growth rate, which is determined by the solution of the FBA model. The Michaelis-Menten kinetic uptake model is given by

$$\begin{aligned} v_{\mathrm {NO_3}} = v_{\mathrm {NO_3,max}} \frac{c_{\mathrm {NO_3}}}{K_\mathrm{m} + c_{\mathrm {NO_3}}} \left( 1 - \frac{\omega _{\mathrm {N}}}{\omega _{\mathrm {N,max}}} \right) , \end{aligned}$$

where the second term models the saturation, i.e., when the nitrogen quota reaches its maximum value $\omega _{\mathrm {N,max}}$. It is assumed that the intracellular nitrogen is stored in form of nitrate. The maximal nitrogen quota is considered as a model parameter. The value for the maximal nitrate uptake rate $v_{\mathrm {NO_3,max}}$ is 0.0309 ${\rm g}_{\mathrm {NO_3}}/{\rm g}_\mathrm{Dw}/{\rm h}$, the value of the half-saturation constant $K_\mathrm{m}$ is 0.0013 ${\rm g}_{\mathrm {NO_3}}/{\rm L}$ are based on [6].

The complete system of equations for the dynamic photobioreactor model are

$$\begin{aligned} \dot{x}&= \upmu x, \end{aligned}$$

$$\begin{aligned} \dot{c}_{\mathrm {NO_3}}&= -v_{\mathrm {NO_3}} x, \end{aligned}$$

$$\begin{aligned} \dot{\omega }_{\mathrm {Chl}}&= m_{\mathrm {Chl}}v_{\mathrm {Chl}} - \upmu \omega _{\mathrm {Chl}}, \end{aligned}$$

$$\begin{aligned} \dot{\omega }_{\mathrm {Car}}&= m_{\mathrm {Car}} v_{\mathrm {Car}} - \upmu \omega _{\mathrm {Car}}, \end{aligned}$$

$$\begin{aligned} \dot{\omega }_{\mathrm {N}}&= \frac{m_{\mathrm {N}}}{m_{\mathrm {NO_3}}}v_{\mathrm {NO_3}} -\mu \omega _{\mathrm {N}}, \end{aligned}$$

where $\mu , v_{\mathrm {Car}},v_{\mathrm {Chl}}$ and $v_{\mathrm {NO_3,met}}$ are determined by the FBA model via lexicographic optimization as described in [9]. Specifically, DFBAlab requires the specification of lexicographic optimization objectives to avoid the common problem of non-unique exchange fluxes that render the ODE system impossible to integrate. The hierarchy (or lexicographic ordering) chosen is shown in Table 1. The ordering is motivated by the assumption that the primary objective is the maximization of growth, then the remaining resources prioritized based on their importance to maintain metabolic activity, and finally minimizing the uptake of nutrients under the constraints that all previous objectives are met.

**Table 1:** Priority list order for the lexicographic linear programs

Level	Objective
1	Maximize biomass production (autotrophic)
2	Minimize chlorophyll accumulation
3	Minimize $\upbeta$-carotene accumulation
4	Minimize nitrate metabolization flux

### Parameter estimation

**Fig. 2:**
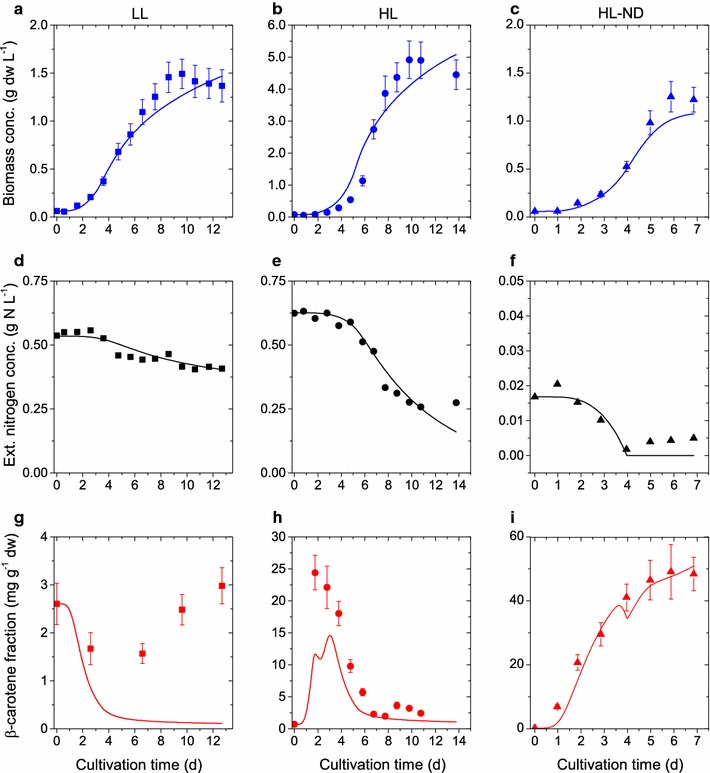
Comparison of simulation results and calibration data for biomass growth, nitrogen consumption and $\upbeta$-carotene accumulation: Simulation results (*lines*), experimental data (*circles*) and* error bars* (two standard deviations) under three different conditions. *First column* (**a**, **d**, **g**) low-light, nitrogen-repleted conditions (LL). *Second column* (**b**, **e**, **h**) high-light, nitrogen-repleted conditions (HL). *Third column* (**c**, **f**, **i**) high-light, nitrogen-depleted conditions (HL-ND)

**Fig. 3:**
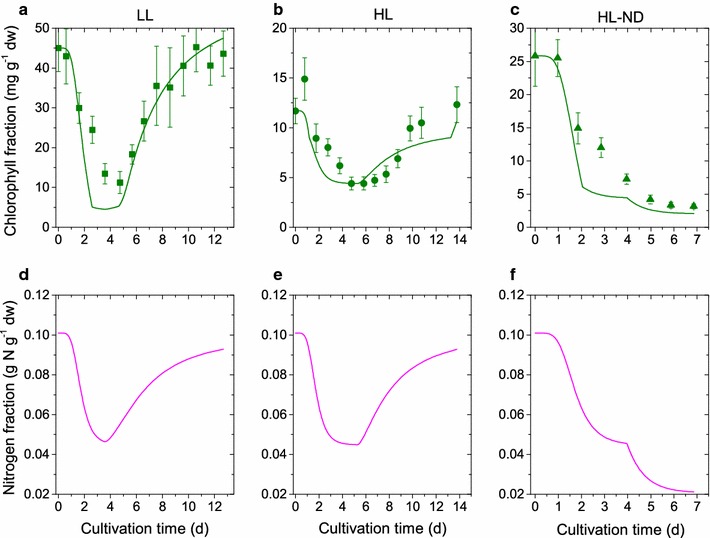
Comparison of simulation results and calibration data for chlorophyll fraction and internal nitrogen cell quota: Simulation results (*lines*), experimental data (*symbols*) and* error bars* under three different conditions. *First row* good fit model for chlorophyll fraction. *Second row* prediction without measurements of nitrogen quota. *First column* (**a**, **d**) low-light, nitrogen-replete conditions (LL). *Second column* (**b**, **e**) high-light, nitrogen-replete conditions (HL). *Third column* (**c**, **f**) high-light, nitrogen-depleted conditions (HL-ND)

The data published in [6] represent three experimental conditions. The first cultivation was performed under low-light, nitrogen-replete conditions (LL) under which neither light nor nutrient stress is induced. LL-conditions lead to no $\upbeta$-carotene accumulation and relatively low biomass production. The second cultivation was performed under high-light and nitrogen-replete conditions (HL) under which only light stress is induced. HL-conditions lead to high biomass production with temporary $\upbeta$-carotene accumulation. The third cultivation was performed under high-light, nitrogen-depleted conditions (HL-ND) under which light and nutrient stress are induced. HL-ND conditions lead to relatively low biomass production, but high $\upbeta$-carotene accumulation. We used these data from standard batch cultivation to estimate an initial set of model parameters. Parameter estimation is performed by minimizing the cost function

$$\begin{aligned} J(\mathbf {p}) = \chi ^2(\mathbf {p}) + \alpha \Theta (\mathbf {p})^2, \end{aligned}$$

where the residual sum of squares $\chi ^2$ is given by Eq. 1, $\Theta$ is the penalty function returned by DFBAlab and $\alpha$ is a positive weighting factor. The penalty function is included such that the optimal solution of the parameter estimation is a feasible DFBA simulation. It is possible that for arbitrary parameter values, the linear programs embedded in the DFBA model are not feasible at every time instance during a batch simulation. DFBAlab provides a penalty value that is zero if the simulation is feasible and strictly greater than zero otherwise (see [9] for further details). Hence, a large weighting factor ensures that infeasible DFBA simulations are penalized in the optimization problem. Different algorithms to solve the parameter estimation problem have been considered. Gradient-based methods performed very poorly and did not converge to a local optimal solution (Table 2). One explanation for this is the fact that the optimization problem is non-smooth, which means that the gradient is potentially undefined. Therefore numerical approximation of gradient information through finite differences potentially does not provide the correct information and causes the algorithm to fail. Development of new theory and algorithms based on non-smooth analysis is necessary to address this issue properly. Heuristic optimization algorithms for the parameter estimation problem that are not based on gradient information were able to provide a good solution, even though no guarantee of optimality could be issued. The best solution was found by a genetic algorithm using the *ga* function in MATLAB. Computations were performed on the high performance computing cluster at the Max Planck Institute for Dynamics of Complex Technical Systems. The results of the parameter estimation are summarized in Table 2. The time evolution of the model solutions, with parameters values given in Table 2, compared to the experimental data under different light and nutrient conditions are illustrated in Figs. 2 and 3. The model simulations agree very well with the experimental data for all measured outputs.

**Table 2:** Best parameter estimates for batch culture data (initial model: value I) and additionally optimized fed-batch culture data (refined model: value II)

Symbol	Description	Value I	Value II	Units
$\alpha _{\mathrm {0}}$	Offset parameter $\upbeta$-carotene synthesis	4.2 $\times 10^{-2}$	6.5 $\times 10^{-2}$	–
$\alpha _{\mathrm {1}}$	Gain parameter $\upbeta$-carotene synthesis	3.47 $\times 10^{-5}$	7 $\times 10^{-5}$	m$^2$ s $\upmu$mol$^{-1}$ photons
$\gamma _{{\mathrm {max}}}$	Max. intracell. chlorophyll to nitrate ratio	0.69	0.75	g Chl g$^{-1}$ N
$\eta _{\mathrm {0}}$	Offset parameter for chlorophyll specific eff.	8 $\times 10^{-4}$	0	–
$\eta _{\mathrm {1}}$	Gain parameter for chlorophyll specific eff.	3.2	3.2	–
$\lambda$	Duration of the lag phase	1.37	1.37	d
$\omega _{\mathrm {N,min}}$	Min. nitrogen cell quota	0.021	0.021	g N g$^{-1}\,$dw
$\omega _{\mathrm {N,max}}$	Max. nitrogen cell quota	0.101	0.101	g N g$^{-1}\,$dw
$\bar{E}_{\mathrm {x,A}}$	Crit. light int. for $\upbeta$-carotene synthesis	32	420	$\upmu$mol photons g$^{-1}$ dw h$^{-1}$
$\bar{E}_{\mathrm {sat}}$	Saturation light int. for chlorophyll to nitrate ratio	75.5	75.5	$\upmu$mol photons m$^{-2}$s$^{-1}$
$K_{\mathrm {E}}$	Inhibition coeff. for chlorophyll to nitrate ratio	12.5	12.5	$\upmu$mol photons m$^{-2}$s$^{-1}$
$K_{\mathrm {m}}$	Michaelis–Menten coeff. for NO$_{3}$ uptake	0.0013	0.0013	g NO$_{3}$ L$^{-1}$
$n_{\mathrm {Car}}$	Hill coefficient for $\upbeta$-carotene synthesis	4	2	–
*ngam*	Non-growth associated maintenance	0.183	0.183	mmol g$^{-1}$ dw h$^{-1}$
$v_{\mathrm {Car,max}}$		3 $\times 10^{-3}$	18 $\times 10^{-3}$	mmol g$^{-1}$ dw h$^{-1}$
${v}_{\mathrm {NO_3,max}}$	Max. nitrogen uptake Max. $\upbeta$-carotene synthesis rate rate	0.0309	0.0309	g NO$_{3}$ g$^{-1}$dw h$^{-1}$ L$^{-1}$
$v_{\mathrm {NO_3,met,max}}$	Max. nitrogen assimilation flux	0.19	0.19	mmol g$^{-1}$ dw h$^{-1}$
$x_{\mathrm {eff}}$	Photon efficiency	0.0375	0.0375	–
$x_{\mathrm {dim}}$	Unit conversion factor	3.6	3.6	L g$^{-1}$
$\eta _{\mathrm {s}}$	Smoothing factor	4000	40	–

### Model validation

**Fig. 4:**
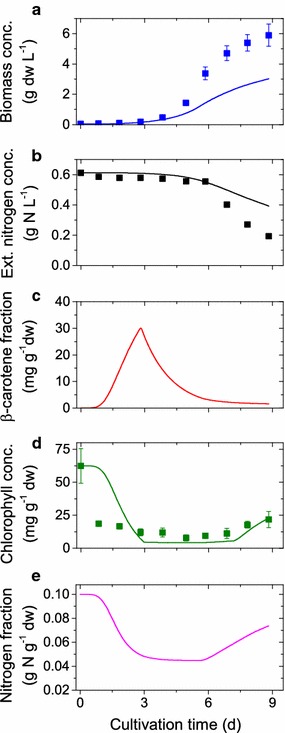
Comparison of simulation results and validation data: Simulation results (*lines*), experimental data (*symbols*) and* error bars* of the validation runs under nitrogen-replete conditions with medium light intensity. Importantly, data were not used to calibrate the model

The model predictions are validated through comparison with an independent set of experimental data that was not used for the parameter estimation. The validation experiment was conducted in the INFORS photobioreactor (see “Methods” section for details) under medium light (ML) and nitrogen-replete conditions. The model prediction and validation data are illustrated in Fig. 4. An overview of the experimental conditions used to generate and validate the computational model can be found in Table 3.

**Table 3:** Overview of the experimental conditions for generating the computational model

Purpose	Abbreviation	Reactor	Light $\upmu$mol photons m$^{-2}$s$^{-1}$	Nitrate gNO$_3$ L$^{-1}$	Figure(s)
Model calibration	LL	PSI	178	2.37	2, 3
Model calibration	HL	PSI	1950	2.76	2, 3
Model calibration	HL-ND	PSI	1950	0.07	2, 3
Model validation	ML	INFORS	250	2.71	4

### Analysis of the model parameters

**Fig. 5:**
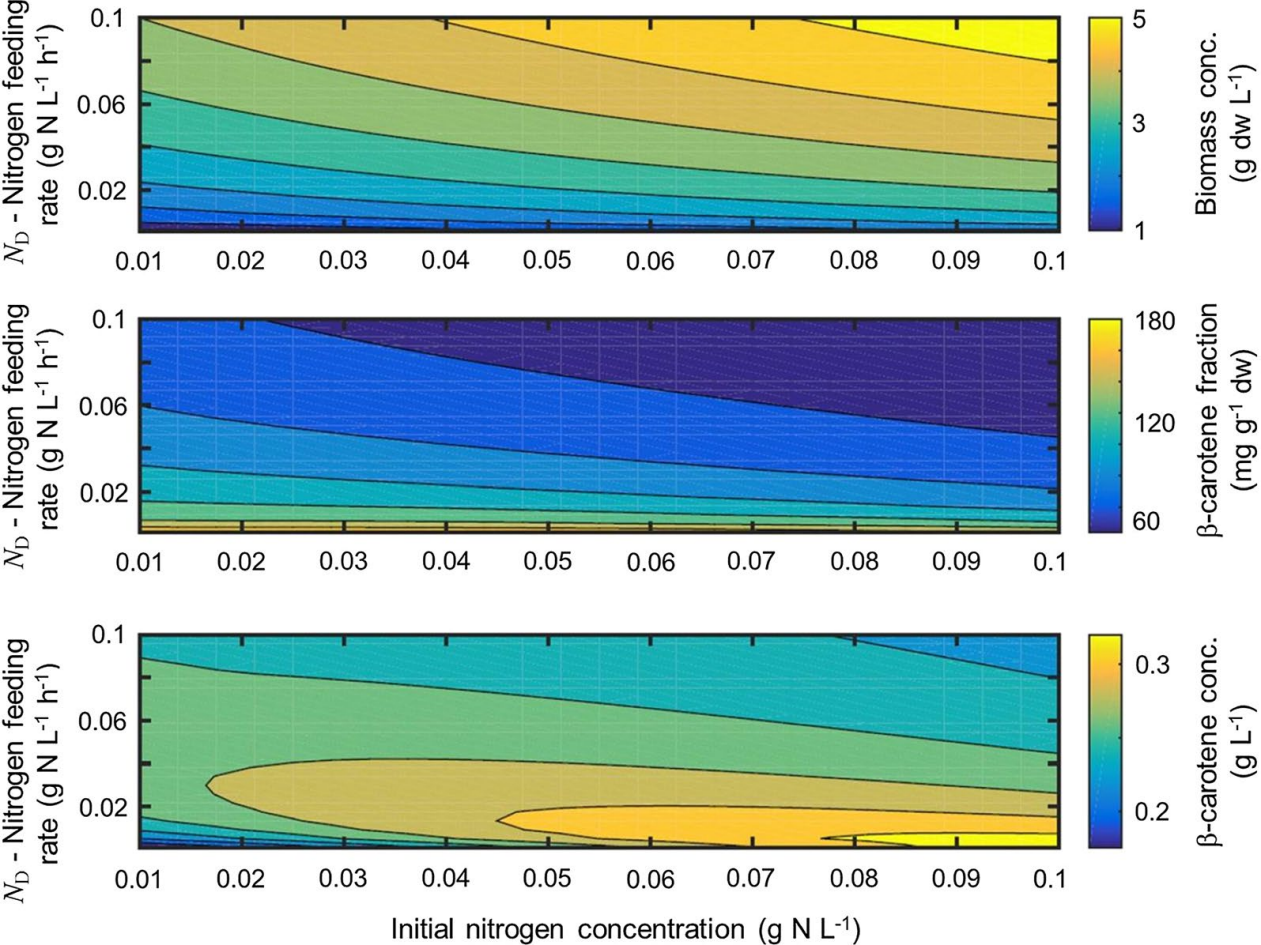
Influence of inoculum nitrate concentration and nitrogen feeding factor $N_D$ on biomass (*top*), $\upbeta$-carotene fraction (*middle*) and total $\upbeta$-carotene amount (*bottom*). For the experiment, inoculum nitrate concentration was at $0.08\,{\rm g}/{\rm L}$. We thus chose $N_D=0.02\,{\rm g}_{\mathrm {N}}{\rm L}^{-1}{\rm h}^{-1}$

The total number of model parameters is 20, which is comparable to existing dynamic kinetic models, for example [19] with 10 model parameters. A more systematic parameter identification to determine which parameters can be identified based on the output measurements would be necessary to potentially reduce the number of parameters. The light-limited growth strongly depends on the efficiency factor $\eta$. A linear relationship between light-limited photosynthesis rate and the chlorophyll fraction has also been proposed in [19]. In [19] it was furthermore shown that this is independent of the nitrogen cell quota. The model also predicts a constant chlorophyll to nitrogen ratio of 0.09 ${\rm g}_{\mathrm {Chl}}/{\rm g}_{\mathrm {N}}$ for irradiance higher than 75.5 μmol/m^2^/s, this value is within the range of values reported in [14, 15] for different microalgae species under nutrient-replete conditions. Stress conditions under which $\upbeta$-carotene synthesis is observed can be quantitatively described by the $\upbeta$-carotene submodel. The best parameter values for the linear relationship between nitrogen cell quota and biomass specific light flux density are $\alpha _0 = 0.042$, $\alpha _1=3.5\times 10^{-5}$ for the initial data set and $\alpha _0 = 0.065$, $\alpha _1=7\times 10^{-5}$ after the fed-batch optimization (parameter set II). Note that under low light conditions no $\upbeta$-carotene is synthesized. For the parameter set II, at the lowest value of the average biomass specific light intensity ($\bar{E}_x= 32\,{\rm mE}/{\rm g}_{\rm dw}/{\rm h}$) any nitrogen cell quota less than $0.06722\,{\rm g}_{\mathrm {N}}/{\rm g}_{\rm dw}$ is predicted to induce stress. These values are relatively low, given that the minimal nitrogen cell quota is estimated to be $0.02\,{\rm g}_{\mathrm {N}}/{\rm g}_{\rm dw}$ resulting in a small operation window for optimized nitrogen feeding (see fed-batch optimization Fig. 5).

### Limitations of the model

In general we observe, that the model predictions agree reasonably well with the data. The model mismatch in the final phase under medium light conditions (Fig. 4) is most likely due to a mismatch in the photosynthetic efficiency represented by the parameters $\eta _{0}$ and $\eta _{1}$, see Eq. 2 and Table 2, which results from the shorter light path of the INFORS reactor used for the validation run. However note that this mismatch can be reduced by re-adjusting the parameters. This also has to be done for any new reactor-species configuration before meaningful model-based product optimization can be performed.

### Model-based fed-batch optimization

As can be seen from Figs. 2 and 3, *D. salina*  grows well for low to high light conditions at sufficient supply of nitrate. In the stress condition of high light and low nitrogen supply, we observe a strong $\upbeta$-carotene accumulation, however, the growth rate is small and on the order of low light conditions. Given a validated computational model of *D. salina*, we planned an optimal fed-batch cultivation to trade-off growth and $\upbeta$-carotene accumulation optimally, where two input variables (i) light and (ii) nitrogen were optimized. In Fig. 1 we illustrated our $\upbeta$-carotene synthesis model. As can be seen, light and nitrogen are interrelated (see also Eq. 3). We therefore simplified the optimization in a first step via exertion of a fixed light stress per cell. After inoculation an initial 1 day adaptation phase at incident light intensity $E_{0}=100\,\upmu$mol photons m$^{-2}$s$^{-1}$ was performed followed by the light intensity profile

$$\begin{aligned} E_{0,\mathrm{opt}}(t)=I_{0} x. \end{aligned}$$

The factor $I_0=3000\,\upmu$mol photons m$^{-2}$s$^{-1}$ L g$^{-1}$ dw was motivated by natural sunlight conditions in open pond operation systems. Note that until $x=1\,{\rm g}_\mathrm{dw}/{\rm L}$, the light intensity profile can sustain a fixed incident light stress per cell. With this light feed we identified an optimal nitrogen feeding profile. The nitrogen feeding profile was parameterized as a Hill function with Hill coefficient $n_D$ and nitrogen fraction ratio $\omega _{\mathrm {N,min}}/\omega _{\mathrm {N}}(t)$,

$$\begin{aligned} N_{{\rm feed},{\rm opt}}(t)=N_D\frac{\left( \omega _{\mathrm {N,min}}/\omega _{\mathrm {N}}(t)\right) ^{n_D}}{1+\left( \omega _{\mathrm {N,min}}/\omega _{\mathrm {N}}(t)\right) ^{n_D}}. \end{aligned}$$

**Fig. 6:**
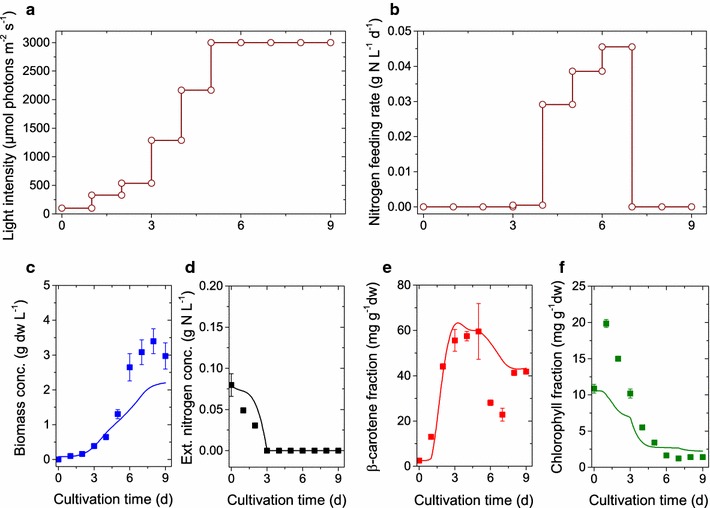
Dynamics of the optimized fed-batch run: Simulations results with refined model structure and parameter set (*lines*), experimental data (*symbols*) and* error bars*. The two *top panels* show the suggested and applied nutrient feeds

We set $n_D=10$ and $\omega _{\mathrm {N,min}}=0.02$. The chosen S-shape of the nitrogen feed parameterization seems suitable for a growing culture. For fixed light stress, the nitrogen quota is the most dominating factor for $\upbeta$-carotene synthesis. Given these two feed parameterizations, we used the model to analyze the behavior of biomass growth and $\upbeta$-carotene accumulation for a fed-batch time of 9 days. As can be seen in Fig. 5 regimes for optimal growth are suboptimal for $\upbeta$-carotene accumulation and vice versa. Fig. 5 also depicts the $\upbeta$-carotene concentration in the reactor, i.e. $\upbeta =\omega _{\mathrm {car}}x$. The optimal design point can be chosen along a line of different combinations of internal nitrogen and nitrogen feeding factor $N_D$. Since the internal nitrogen at the beginning of the fed-batch cultivation is determined by the inoculum, the nitrogen feeding factor was determined to $N_D=0.02\,{\rm g}_{\mathrm {N}}{\rm L}^{-1}{\rm h}^{-1}$. The resulting input profiles were realized in an experimental run, where both profiles have been discretized in daily integrated applications (see. Fig. 6b). During the experimental run the algae population grew faster than the one used in the preceding batch experiments. Already after 7 days of optimized cultivation we reached a saturation in the maximal light stress and the $\upbeta$-carotene amount started to decline (Fig. 6). Inducing an additional stress via nitrogen depletion, the $\upbeta$-carotene fraction could again be raised. At the optimal harvesting time point, we found a $\upbeta$-carotene density of 0.140 g/L at a fraction of 0.06 ${\rm g}_{\mathrm {car}}/{\rm g}_{\rm dw}$ and biomass of 3.4 ${\rm g}_{\rm dw}/{\rm L}$. This is about 2.1 times more $\upbeta$-carotene compared to the standard HL-ND batch (see Table 4; Figs. 2 c, f, i and 3 c, f).

**Table 4:** Performance of the optimized fed-batch run compared to the standard batch conditions (HL-ND) conducted in the PSI reactor

Property	Unit	Standard batch	Optimized fed-batch
Max. biomass density	${\rm g}_\mathrm{dw}\,{\rm L}^{-1}$	1.38	3.40
Max. $\upbeta$-carotene density	${\rm mg}\,{\rm L}^{-1}$	67.73	140.06
Max. $\upbeta$-carotene fraction	${\rm mg}\,{\rm g}_{\rm dw}^{-1}$	49.09	59.53
Max. $\upbeta$-carotene productivity	${\rm mg}\,{\rm L}^{-1}{\rm d}^{-1}$	11.53	17.48

## Conclusion

An extension of the DFBA formulation that accounts for intracellular accumulation of valuable metabolites has been presented. For the accumulation of $\upbeta$-carotene in *D. salina*, a detailed model has been developed that accurately predicts synthesis of $\upbeta$-carotene under various light and nutrient conditions. The model was validated using an independent set of experimental data. The model was further used to optimize a fed-batch culture with respect to light and nitrogen feeding. As we have shown, the model-based fed-batch optimization doubled $\upbeta$-carotene concentration in the reactor. The benefit of the structured model formulation based on metabolic network information is that it provides quantitative prediction of the conditions under which increased $\upbeta$-carotene synthesis is observed. It was illustrated that inclusion of existing detailed metabolic models within process models shifts the modeling efforts towards determining kinetic sub-models that best describe particular aspects such as $\upbeta$-carotene accumulation. Future work aims at developing new methods to provide sensitivity information for this class of non-smooth dynamic systems. Deterministic optimization algorithm with guaranteed convergence rates can then use the sensitivity information to solve the dynamic optimization problem. Overall, this development is a necessary step towards a rational design procedure for biotechnological processes.
